# Predictors of physical activity in older adults early in an emergency hospital admission: a prospective cohort study

**DOI:** 10.1186/s12877-020-01562-3

**Published:** 2020-05-18

**Authors:** Peter Hartley, Amanda L. DeWitt, Faye Forsyth, Roman Romero-Ortuno, Christi Deaton

**Affiliations:** 1 Primary Care Unit, Department of Public Health and Primary Care, University of Cambridge, Cambridge, UK; 2 Department of Physiotherapy, Cambridge University Hospital NHS Foundation Trust, Cambridge, UK; 3 Forvie Site, University of Cambridge School of Clinical Medicine, Cambridge Biomedical Campus, Cambridge, CB2 0SR UK; 4 Department of Medicine for the Elderly, Cambridge University Hospital NHS Foundation Trust, Cambridge, UK; 5 Discipline of Medical Gerontology, Trinity College Dublin, Mercer’s Institute for Successful Ageing, St James’s Hospital, Dublin, Ireland

**Keywords:** Aged, Hospital, Physical activity, Accelerometers, Functional mobility, Illness severity

## Abstract

**Background:**

Reduced mobility may be responsible for functional decline and acute sarcopenia in older hospitalised patients. The drivers of reduced in-hospital mobility are poorly understood, especially during the early phase of acute hospitalisation. We investigated predictors of in-hospital activity during a 24-h period in the first 48 h of hospital admission in older adults.

**Methods:**

This was a secondary analysis of a prospective repeated measures cohort study. Participants aged 75 years or older were recruited within the first 24 h of admission. At recruitment, patients underwent a baseline assessment including measurements of pre-morbid functional mobility, cognition, frailty, falls efficacy, co-morbidity, acute illness severity, knee extension strength and grip strength, and consented to wear accelerometers to measure physical activity during the first 7 days (or until discharge if earlier). In-hospital physical activity was defined as the amount of upright time (standing or walking). To examine the predictors of physical activity, we limited the analysis to the first 24 h of recording to maximise the sample size as due to discharge from hospital there was daily attrition. We used a best subset analysis including all baseline measures. The optimal model was defined by having the lowest Bayesian information criterion in the best-subset analyses. The model specified a maximum of 5 covariates and used an exhaustive search.

**Results:**

Seventy participants were recruited but eight were excluded from the final analysis due to lack of accelerometer data within the first 24 h after recruitment. Patients spent a median of 0.50 h (IQR: 0.21; 1.43) standing or walking. The optimal model selected the following covariates: functional mobility as measured by the de Morton Mobility Index and two measures of illness severity, the National Early Warning Score, and serum C-reactive protein.

**Conclusions:**

Physical activity, particularly in the acute phase of hospitalisation, is very low in older adults. The association between illness severity and physical activity may be explained by symptoms of acute illness being barriers to activity. Interdisciplinary approaches are required to identify early mobilisation opportunities.

## Background

Unnecessarily reduced physical activity during hospitalisation may be part responsible for hospital-associated functional decline and acute sarcopenia in older patients [1, 2]. Higher levels of physical activity whilst in hospital have been shown to correlate negatively with risk of death [3], length of stay [4], and functional decline [5], and positively with likelihood of discharge home [6].

Reports of the amount of physical activity in hospitalised older adults have varied, with median time spent standing or walking per day in hospital reported as being between 45 and 83 min [7–9]. Although this is considered ‘low’ it is unclear what the optimal level of activity is, and there are no universally accepted clinical guidelines in this area [10].

There has been an increase in clinical [11] and research focus [6, 12, 13] to increase mobilisation on acute medical and geriatric wards, with an emphasis on protocols designed to improve the *early* and regular implementation of physical mobility activities to improve the health outcomes of hospitalised older people [14].

Previous studies from Denmark [7, 9], Norway [15] and the United States of America [10] have found pre-morbid levels of functional mobility [7, 9, 10, 15], cognitive ability [7] and acute illness severity [10] to be important determinants of ambulation in hospitalised older adults. However, the drivers of reduced in-hospital mobility are poorly understood, especially during the early phase of acute hospitalisation. We investigated clinical predictors of in-hospital activity during the first 24 h of hospital admission in older adults in the United Kingdom (UK) using the innovative method of best-subset analysis.

## Method

### Setting

Patients were recruited from Cambridge University Hospital NHS Foundation Trust (CUH), a large tertiary university hospital in England with over 1000 beds. In 2018 CUH had 158,399 visits to the emergency department (ED), with 44,120 emergency admissions [16].

### Study design

This was a secondary analysis of a prospective repeated measures cohort study [17]. Ethical approval was granted by the London Queen Square Research Ethics Committee (17/LO/1817). All participants provided written informed consent.

### Patient and public involvement (PPI)

Before the study began, a PPI panel was convened. The study design reflects amendments and changes suggested by the panel and patients. The panel also reviewed the final versions of the participant information sheet and consent form.

### Sample

We included patients admitted to CUH over an 11-month period (Jan 2018 to Dec 2018), who were aged 75 years or older, experiencing an unplanned hospitalisation (i.e. non-elective), able to give informed consent and expected to be hospitalised for at least 48 h. *Exclusion criteria:* admitted more than 24 h before recruitment; unable to provide informed consent based on an assessment of the patient’s mental capacity (a diagnosis of dementia was not in itself an exclusion criterion); receiving end-of-life care or treatment for diagnosed cancer; inability to cooperate in muscle-strength testing (e.g. unable to sit in chair, or skin integrity problem contraindicating the use of a hand-held dynamometer); transferred to or from the intensive care unit; bed-bound or requiring a hoist to transfer from bed to chair within the 2 weeks before hospitalisation; allergy to adhesive dressings; or if the clinical team had any other concerns regarding skin integrity around the proposed accelerometer sites. Sampling was convenience-based in that most screening took place Monday to Friday, 8:00 to 18:00. On recruitment days all patients over the age of 75 and admitted within the last 24 h were consecutively screened by a member of the clinical team. Patients who met the inclusion criteria were approached regarding participation.

### Sample size

The sample size was based pragmatically on maximising the number of recruits over an 11-month period.

### Procedures

Participants were recruited during the first 24 h of their hospital admission. Recruitment was performed by PH (a physiotherapist with 10 years of clinical experience) or a research nurse, all assessments of patient capacity to consent were made by PH. At recruitment, a series of baseline measurements were taken, and the participants were fitted with accelerometers to measure physical activity.

### Measurements

All measurements were taken by an experienced physiotherapist. Baseline measurements consisted of: age, sex, weight, frailty, acute illness severity, co-morbidity burden, falls efficacy, cognition, a self-reported measure of functional ability, a measure of functional mobility and objective physical activity levels via accelerometery.

The objective level of in-hospital physical activity was recorded using wearable accelerometers (AX3, Activity, Newcastle upon Tyne, UK), mounted mid-thigh and at the ankle, attached with adhesive dressings [18]. Using a validated method, data collected included the amount of time in a lying position, sitting position, standing position and walking [18]. The accelerometers were worn by participants after they provided informed consent, and were removed on day 7 or discharge, whichever was earliest. Accelerometer sites were checked daily and re-dressed if the dressing was losing adhesion.

The Survey of Health, Ageing and Retirement in Europe Frailty Instrument (SHARE-FI) tool was used to measure frailty. The SHARE-FI tool is a well validated and simple measurement of physical frailty [19]. Five SHARE variables approximating Fried’s frailty phenotype definition are used: fatigue, loss of appetite, grip strength, functional difficulties and physical activity. Scores range between − 2.7 and 13.4 (with 13.4 indicating the most severely frail) [19]. As it is routinely measured as part of clinical care, the Clinical Frailty Scale (CFS) score was also recorded [20]. The scoring of the CFS is based on a global assessment of patients’ comorbidity symptoms, cognition, level of physical activity and dependency on activities of daily living. The possible scores range from 1 (very fit) to 9 (terminally ill).

To measure acute illness severity, we used serum C-reactive protein (CRP) levels, and the National Early Warning Score (NEWS), both of which are routinely collected on admission. CRP is an acute phase-reactant protein released in response to injury, infection or inflammation and is a recognised clinical measure of illness severity [9, 10]. This was collected only for clinical reasons, therefore if CRP was not measured on admission but on day 1 of the study, then the day 1 value was used. The half-life of serum CRP in humans is approximately 19 h [11, 12].

The NEWS was devised by the Royal College of Physicians of London to standardise the assessment and response to acute illness [21] and has been extensively validated [22].

The Charlson Comorbidity Index (CCI) is a method for classifying comorbid conditions for use as a prognostic indicator [23]. The CCI is based on patients’ diagnoses as coded by the World Health Organization’s International Classification of Diseases (10th version).

Falls efficacy is defined as self-perceived confidence in engaging in activities of daily living without falling [24]. The FES-I is a reliable and validated measure in older adults [25].

The Mini-ACE is a 30-point scale used to detect cognitive impairment [26]. The Mini-ACE has been reported to have higher sensitivity and higher ceiling effect than the Mini-Mental State Examination [26] . A score of 25 or less is suggestive of cognitive impairment [26] .

Self-reported general functional ability was measured using the Barthel Index: a 10-item ordinal scale (0–100) of functional independence with activities of daily living, where a score of 100 represents a high level of functional independence [27]. The participants were asked at baseline assessment to base their answers on their functional ability two weeks before admission.

The DEMMI is a 100-point ordinal scale for the assessment of mobility in older acute medical patients [28]. It consists of 15 items ranging from assessing bed mobility to high levels of dynamic balance. A score of 100 represents a high level of functional mobility [28]. The DEMMI provides interval level measurement and does not have floor or ceiling effects in the acute hospital setting.

Knee-extensor HHD (using the microFET 2, Hoggan Scientific, Salt Lake City, Utah) was measured in participants seated with their knee at 90° with the HDD perpendicular to the leg above the superior border of lateral malleolus; patients were asked to push against it with maximum effort [29]. The HHD was tethered to a stationary object whilst the researcher held it in place to prevent the leg from moving. Force was converted to torque (Nm) by multiplying the result by the distance between the superior border of the lateral malleolus and superior border of the lateral femoral epicondyle. Grip strength HHD (using the JAMAR device, Sammons Preston, Bolingbrook, Illinois) was measured with participants seated with elbow at 90° and wrist in neutral position; participants were instructed to squeeze as hard as possible for a few seconds [30]. With both knee-extensor and grip strength dynamometry readings, participants were asked to repeat the procedure three times on both their left and right sides, the highest force measurements from each side were then averaged to provide the score.

In-hospital physical activity was defined as the amount of upright time (standing or walking).

## Analysis

Data were analysed with R software [31]. Continuous variables were described as median or inter-quartile range (IQR), and categorical variables as count and percentage. The analysis was limited to the first 24 h of measured activity due to attrition of data as patients were discharged from hospital.

To examine the predictors of physical activity in the first 24 h of study participation we used a best subset analysis using the ‘leaps’ R package [32]. To comply with the assumption of normality of residuals, upright time was transformed using a base-10 logarithm transformation. The model specified a maximum of 5 covariates and used an exhaustive search. To assess for overfitting of the data, the Bayesian information criterion (BIC) was extracted. We also performed k-fold cross validation (k = 10) to predict the different models’ ability to generalise to independent data sets.

## Results

Seventy participants were recruited but eight were excluded from the final analysis due to lack of accelerometer data within the first 24 h. Reasons for missing data included: clinical need for MRI scans and therefore unable to wear accelerometers (*n* = 3), withdrawal (n = 3), contraindicating skin condition (*n* = 1), and device malfunction (n = 1). A further 15 patients only wore one accelerometer on their thigh due to poor skin condition on their lower legs. For these participants it is not possible to differentiate between lying and sitting. Participant characteristics and levels of activity are presented in Table 1. There was a median time of 19.6 h (IQR: 15.9; 22.9) between admission to hospital and baseline assessment.

**Table 1:** Participant Characteristics (*n* = 62)

Characteristics	Summary measure: median (IQR) or count (%)
Female	26 (41.9%)
Age (years)	85.0 (80.2; 87.0)
Weight (kg)	68.7 (56.2; 78.8)
CFS	4.5 (4.0; 5.0)
SHARE-FI	3.4 (1.6; 4.3)
CCI	2.0 (1.0; 3.0)
Falls in last 12 months	1.0 (0.0; 2.0)
Admission CRP (mg/L)	46.0 (8.1; 119.2)
NEWS	2.5 (1.0; 4.0)
Barthel Index (self-reported 2-weeks prior to admission)	90.0 (76.2; 100.0)
DEMMI	41.0 (33.8; 56.0)
FES-I	26.5 (20.2; 33.8)
Mini ACE	27.0 (25.0; 28.8)
Grip strength (kg)	18.2 (14.0; 23.6)
Knee torque (Nm)	43.4 (33.8; 57.8)
Active time (standing or walking) first 24 h of study (hours)	0.50 (0.21; 1.43)

*Abbreviations*: *CFS* Clinical Frailty Scale, *SHARE-FI* Survey of Health, Ageing and Retirement in Europe Frailty Instrument; *CCI* Charlson Comorbidity Index; *CRP* C-reactive protein, *NEWS* National Early Warning Score, *DEMMI* de Morton Mobility Index, *FES-I* Falls Efficacy Scale – International, *Mini-ACE* Mini-Addenbrooke’s Cognitive Assessment

**Fig. 1:**
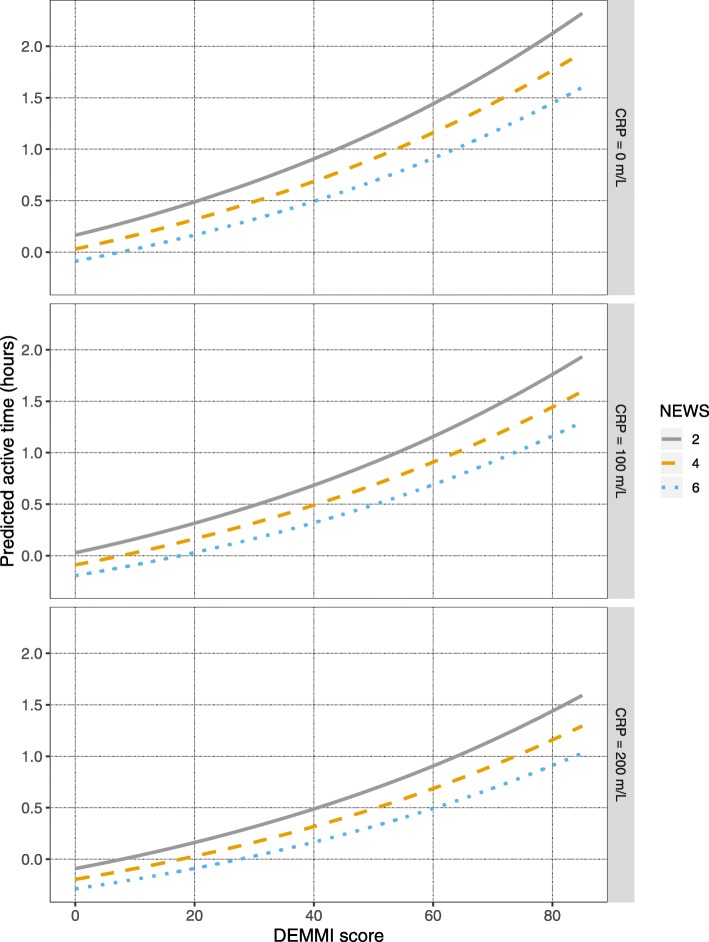
Active time (hours) as predicted by functional mobility and illness severity (back transformed regression). Abbreviations: CRP = C-reactive protein; DEMMI = de Morton Mobility Index; NEWS = National Early Warning Score

The results of the best subset analysis are presented in Table 2; all variables in Table 1 were included in the analysis. According to the BIC, the optimal model for predicting physical activity level on admission was model 3, which used functional mobility (DEMMI) and two measures of illness severity (NEWS and CRP) as the co-variates (see Table 3). Further increasing the number of co-variates only marginally increased the adjusted R^2^ value. Figure 1 illustrates the relationship between the back-transformed sedentary activity and the three independent variables. The DEMMI is plotted as a continuous variable on the x-axis, the NEWS and CRP are depicted at 3 arbitrary levels along their continuous scales, the NEWS score 2, 4 and 6 as separate lines, and CRP level 0 m/L, 100 m/L and 200 m/L as separate facets.

**Table 2:** Results of best subset analysis

Model	Covariates in model	BIC	Adjusted R^**2**^	Cross-validation error
1	DEMMI	−4.65	0.18	0.15
2	DEMMI, NEWS	−8.67	0.27	0.14
**3**	**DEMMI, NEWS, CRP**	**−12.61**	**0.35**	**0.13**
4	DEMMI, NEWS, CRP, CCI	−10.98	0.36	0.14
5	DEMMI, NEWS, CRP, CCI, Sex	−8.26	0.37	0.14

*Abbreviations*: *BIC* Bayesian information criterion, *DEMMI* de Morton Mobility Index, *NEWS* National Early Warning Score, *CRP* C-reactive protein, *CCI* Charlson Comorbidity Index

**Table 3:** Summary of model 3 from best subset analysis

Term	Estimate (base 10 logarithmic transformation)	Lower 95% CI	Upper 95% CI	*P* value
DEMMI	0.01	0.00	0.01	<.001
NEWS	− 0.03	−0.04	− 0.01	.002
CRP	0.00	0.00	0.00	.007

*Abbreviations*: *DEMMI* de Morton Mobility Index, *NEWS* National Early Warning Score, *CRP* C-reactive protein

## Discussion

In this study, patients were active for only 30 min during the first twenty-four hours after recruitment (median time from hospital admission to recruitment was 19.6 h). This study confirms findings from previous studies which indicate a low proportion of time spent being active in the early period following an acute admission [7–9, 15]. The study is important in demonstrating the repeatability of this previous work and in indicating that it is generalisable to a UK population.

The best subset model found functional mobility and illness severity to be most predictive of physical activity during the initial period of hospitalisation, explaining 35% of variance in activity time. Higher levels of functional mobility ability and lower illness severity (lower admission CRP and NEWS) were associated with higher amounts of physical activity. These findings are in keeping with previous research regarding the predictive value of functional mobility [7, 9, 10, 15], and illness severity [10] in hospitalised older people.

The association between illness severity and physical activity may be explained by symptoms of acute illness being barriers to activity. Previous studies have highlighted concerns around mobilising patients who are acutely ill, such as respiratory and cardiovascular instability, or who have indwelling devices, weakness, pain, polypharmacy, sleep disturbance, and reduced nutritional intake [33–35].

Functional mobility scored by the DEMMI is likely to reflect the amount of assistance a person needs in order to mobilise. For example, a person with a score of 38/100 would be expected to be able to perform bed based mobility tasks, require minimal assistance or supervision for transfers in and out of the chair, have adequate balance to sit and stand unsupported and walk short distances with assistance or supervision [28]. The availability of staff to provide assistance is frequently cited as a barrier to activity in hospital [33–35]. The functional mobility scores may also reflect clinicians’ assessments of risk of falls, which may affect clinicians’ behaviour and advice, resulting in reduced patient activity [34]. Indeed, 11% of the cohort described by So and Pierluissi [33] reported being actively discouraged from walking, although whether this was to do with a perceived risk of falling, illness severity or another reason is unclear.

It has been hypothesised that older adults with low falls efficacy may restrict their daily activities [36]. Qualitative research has highlighted fear of falling as a barrier to activity in hospital [33, 34]. Falls efficacy however, was not identified as a top predictor of physical activity in any of the 5 models, nor was their evidence of multicollinearity (indicating that falls efficacy was accurately predicted by functional mobility and illness severity).

In the absence of guidelines regarding optimal levels of physical activity in older hospitalised patients, we are unable to determine whether any participants regardless of functional ability or illness severity were adequately active. However, particularly for the least active patients, this work may challenge the belief held by some that it is the patient’s lack of motivation that limits physical activity in hospital [34]. To increase activity in the least active patients, it is hypothesised in the context of these findings, that more staff able to provide physical assistance and appropriately risk assess the dangers of mobilising or indeed not mobilising acutely sick patients are needed. This approach requires an interdisciplinary collaboration between medical, nursing and therapy personnel, similar to the approach that has been reported in intensive care settings [37].

### Limitations

There are several limitations to this research including the small sample size, and the use of a convenience sampling method. Patients with significant cognitive impairments were excluded from this study for reasons of informed consent. Cognitive impairment has been shown to be common in hospitalised older adults and is associated with in hospital functional decline [38] and reduced activity [7]. Accelerometers were attached prior to functional mobility assessments (scoring of the DEMMI) in the baseline assessment. It is therefore possible that participation in the study affected the patient’s level of activity. However, this baseline assessment replaced the routine assessment performed by a physiotherapist of newly admitted patients. The physiotherapy assessment would routinely measure functional mobility though not necessarily with the DEMMI. It is therefore felt that any effect of participation in the study on activity would be minimal but may affect comparison with settings that do not include a routine physiotherapy assessment.

This is also a secondary data analysis, which therefore limits the potential variables explored regarding this specific research question. There is also no qualitative data included in the analysis, as none was collected. Finally, the accelerometer data does not tell us about the reason for activity, or whether or not assistance was provided.

## Conclusions

Physical activity, particularly in the acute phase of hospitalisation, is very low in older adults. Of the variables included in this study, functional mobility and illness severity were found to be the best predictors of physical activity in this acute phase. Given these findings, to increase physical activity in the least active patients, it is hypothesised that more staff trained to provide physical assistance and able to risk assess mobility appropriately in acutely ill patients are needed. Future research should address recommended levels and type of physical activity required during an acute admission to optimise hospital and functional outcomes.

## Data Availability

The datasets generated and/or analysed during the current study are not publicly available as permission was not gained for this from our participants but are available from the corresponding author on reasonable request.

## Abbreviations

BIC: Bayesian information criterion
CCI: Charlson Comorbidity Index
CFS: Clinical Frailty Scale
CUH: Cambridge University Hospital NHS Foundation Trust
CRP: C-reactive protein
DEMMI: de Morton Mobility Index
DME: Department of Medicine for the Elderly
ED: Emergency Department
FES-I: Falls Efficacy Scale – International
IQR: Inter-quartile range
Mini-ACE: Mini-Addenbrooke’s Cognitive Assessment
NEWS: National Early Warning Score
PPI: Patient and Public Involvement
SHARE-FI: Survey of Health, Ageing and Retirement in Europe Frailty Instrument
